# Development of a Wine Metabolomics Approach for the Authenticity Assessment of Selected Greek Red Wines

**DOI:** 10.3390/molecules26102837

**Published:** 2021-05-11

**Authors:** Alexandros Tzachristas, Marilena E. Dasenaki, Reza Aalizadeh, Nikolaos S. Thomaidis, Charalampos Proestos

**Affiliations:** 1 Laboratory of Food Chemistry, Department of Chemistry, National and Kapodistrian University of Athens, Panepistimiopolis Zografou, 15771 Athens, Greece; A.Tzachristas@chem.uoa.gr (A.T.); harpro@chem.uoa.gr (C.P.); 2 Laboratory of Analytical Chemistry, Department of Chemistry, National and Kapodistrian University of Athens, Panepistimiopolis Zografou, 15771 Athens, Greece; raalizadeh@chem.uoa.gr (R.A.); ntho@chem.uoa.gr (N.S.T.)

**Keywords:** red wine, metabolomics, HRMS, chemometrics, biomarkers

## Abstract

Wine metabolomics constitutes a powerful discipline towards wine authenticity assessment through the simultaneous exploration of multiple classes of compounds in the wine matrix. Over the last decades, wines from autochthonous Greek grape varieties have become increasingly popular among wine connoisseurs, attracting great interest for their authentication and chemical characterization. In this work, 46 red wine samples from *Agiorgitiko* and *Xinomavro* grape varieties were collected from wineries in two important winemaking regions of Greece during two consecutive vintages and analyzed using ultra-high performance liquid chromatography-quadrupole time-of-flight mass spectrometry (UHPLC-QToF-MS). A targeted metabolomics methodology was developed, including the determination and quantification of 28 phenolic compounds from different classes (hydroxycinnamic acids, hydroxybenzoic acids, stilbenes and flavonoids). Moreover, 86 compounds were detected and tentatively identified via a robust suspect screening workflow using an in-house database of 420 wine related compounds. Supervised chemometric techniques were employed to build an accurate and robust model to discriminate between two varieties.

## 1. Introduction

Viticulture and winemaking have been practiced in Greece since antiquity [1]. Nowadays, wine trade is a highly competitive area demanding intriguing and authentic wines. The plethora of autochthonous red and white Greek grape varieties are undoubtedly the spearheads towards this direction. *Agiorgitiko* and *Xinomavro* are considered the two most important Greek red grape varieties, producing high quality wines that attract the attention of wine connoisseurs [2]. *Agiorgitiko* is a polydynamic red grape variety cultivated almost exclusively in Nemea Peloponnese, the largest Protected Designation of Origin (PDO) zone for red wines, consisting of vineyards in altitudes ranging from 200–850 m. *Xinomavro* is regarded as the predominant red variety of Northern Greece, used exclusively for the production of PDO Naoussa wines as well as participating in wines from other important PDO regions.

Quality wines have become an inseparable part of contemporary culture, frequently considered as a sign of social status and well-being, and as such, have gained significant commercial value. Consumers’ perceptions of wine quality often rely on the specific attributes of one or more grape varieties, geographical origin, and vintage year [3]. However, due to its economic importance, multiple instances of wine fraud and mislabeling are frequently reported [3,4], often raising pricing uncertainty issues [5]. Consequently, the development of robust methodologies is required to verify wine identity, thus protecting against fraudulent practices and maintaining consumer confidence.

Authenticity control can become a challenging task due to the presence of multiple viti-vinicultural factors involved in the wine production [6]. Authenticity assessment was traditionally conducted through sensory evaluation, and this approach still applies since it is part of the official controls for the certification of wines from protected regions (Reg EU 34/2019). However, as wine is an easily falsified beverage, thorough chemical investigation is also needed to verify its identity. The wine matrix is of complex nature, containing diverse chemicals which demand sensitive analytical techniques to make the chemical characterization efforts successful [7]. The traditional applications of wet chemistry often provided limited information about the wine chemical constituents. In current days, the emergence of analytical instruments of high separation efficiency, high resolution, and exceptional sensitivity coupled to advanced chemometric techniques [8] has provided a new perspective in wine analysis and authentication. The use of such cutting edge tools has promoted the swift development of the wine metabolomics field, being the comprehensive quantitative and qualitative study of small metabolites (<1 kDa) within the wine matrix [9]. Analytical platforms including Gas Chromatography coupled to mass spectrometry (GC-MS), ultrahigh-performance liquid chromatography-quadrupole time-of-flight high resolution mass spectrometry (UHPLC/Q-ToF-MS), Fourier transform ion cyclotron resonance mass spectrometry (FT-ICR-MS), and ^1^H NMR have been used for novel compound discovery [10,11], wine group characterization and discrimination [12,13,14,15,16] as well storage and ageing process monitoring [17,18,19,20]. 

The use of UHPLC/Q-ToF-MS instrumentation has proven to be particularly potent in wine characterization and varietal and geographical origin discrimination [21,22,23]. For instance, Arapitsas et al. (2020) recently provided a comprehensive study of the metabolome of eleven monovarietal Italian red wines using an untargeted UHPLC/Q-ToF-MS methodology. Undoubtably, untargeted analysis presents an holistic approach on the wine metabolome with multiple applications reported [14,20,24,25]. However the numerous non-reported compounds in LC databases that often complicate the identification process is still considered one of the main challenges of this approach [26]. On the other hand, the application of target [27,28] and suspect screening strategies [21,29] have also been successfully employed to seek for patterns or characteristic markers verifying various aspects of grape/wine identity. Flamini et al. monitored the polyphenol content of thirty-four hybrid grape varieties through suspect screening analysis [29]. In another study, Rosso and co-workers presented a targeted metabolomics workflow based on the calculation of secondary metabolite indexes, to identify the unauthorized use of Primitivo and Negro Amaro grapes in the production of Valpolicella wines [30].

The composition of Greek wines from selected autochthonous cultivars has been partially reported using diverse methodologies. Studies included a characterization of their chemical profile [14,15,16] or authenticity assessment [17,18,19], often focusing on specific classes of compounds. To the best of our knowledge, no wide scope chemical screening strategies, employing LC-HRMS metabolomics, have been previously reported concerning red Greek varietal wines and their authenticity issues. 

The study novelty lies on the evaluation, for the first time, of the potential of LC-HRMS-based wine metabolomics in the chemical characterization and discrimination of wines from Agiogitiko and Xinomavro grape varieties using both targeted and suspect screening methodologies. In this context, the first objective was to characterize industrial samples from these two emblematic Greek grape varieties for the presence of 28 phenolic compounds using authentic standards. The second objective was to screen the wines samples through a previously developed in-house database and subsequently detect and identify as much compounds as possible. The third objective was to explore the dataset generated using multivariate methods of statistical analysis, seek for characteristic marker compounds and build accurate classification and prediction models resulting in varietal discrimination.

## 2. Results and Discussion

### 2.1. Method Validation Results

Method linearity was evaluated providing satisfactory results in all cases, allowing a regression coefficient of determination (R^2^) greater than 0.990.

Regarding intraday precision, the majority of the target metabolites (more than 83%) exhibited %RSD values lower than 10% (Table S3). Moreover, relatively low matrix effects were observed in 26 out of 29 metabolites which displayed matrix effects lower than ±40%. LOQ values ranged from 0.0325 mg/L (quercetin) to 0.283 mg/L (vanillic Acid). All the results of the method validation are presented in Table S3 of the ESM.

### 2.2. Target Screening

The concentrations of the target compounds detected in red wines from *Agiorgitiko* and *Xinomavro* grape varieties is presented in Table 1.

Shapiro and Levene tests were conducted to evaluate normality assumptions for univariate analysis (Table S4 of the ESM). These criteria were met in only three cases (gallic, gentisic, and taxifolin). Therefore, Wilcoxon–Mann–Whitney non-parametric tests were used to compare metabolite concentrations between the wines from the two varieties. A correction for multiple tests (Bonferroni adjustment) was applied to adjust the *p*-value of the target compounds evaluated in the analysis. Differences were considered as significant when p_adj < 0.05. Values reported herein are in a general accordance with ranges reported in phenol explorer (www.phenol-explorer.eu; accessed on 10 April 2021) and previously published literature regarding Greek wines [31,32,33].

### 2.3. Suspect Screening Results

A total of 86 compounds from the in-house suspect screening database were detected and tentatively identified in the samples. High mass accuracy was observed (mass error less than 1.5 mDa) and acceptable isotopic pattern fit values (below 100 mSigma). Peak score values (peak area to peak intensity ratio) ranged from 6.2 to 19.9 for all compounds. All the identified compounds were also evaluated based on MS/MS data and retention time plausibility. Moreover, 78 out of the 86 detected compounds were inside the applicability domain of the model and the difference between predicted and experimental retention times did not exceed 1 min for the 65.4% and 2 min for the 93.6% of the suspected compounds. An overview of all compounds (target and suspect) found present in the wine samples (presence in at least 80% of red samples of each variety) is given in Table S5 of the ESM. 

HRMS metabolomics provide new perspectives and limit the boundaries in wine analysis [8]. Recent works focus on either targeted or comprehensive characterization approaches of the wine metabolome and its related processes [34]. Nevertheless, a significant number of the global varietal wealth has not been yet characterized in detail. To the best of our knowledge, this is the first time that a thorough characterization of Greek red wines from *Agiorgitiko* and *Xinomavro* varieties, employing HRMS metabolomics, is reported indicating their high quality and nutritional value.

### 2.4. Statistical Analysis

#### 2.4.1. Data Processing

**Figure 1:**
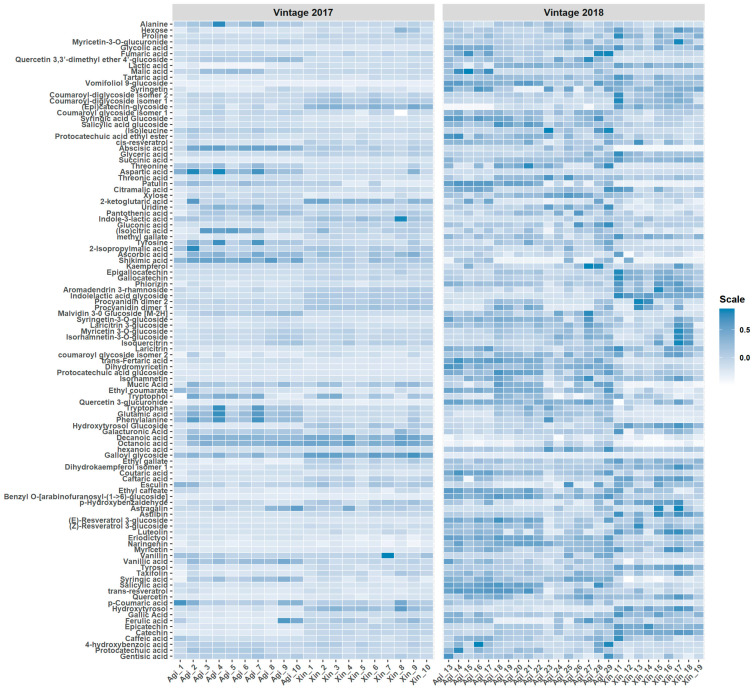
Heatmap of the relative abundances of target and suspect screening compounds. Samples names “Agi…” and “Xin…” correspond to Agiorgitiko and Xinomavro samples, respectively. Row-wise normalization (range scaling) was performed.

A dataset containing peak areas of both target and suspect screening variables normalized to the IS peak area for overall 108 compounds in 46 samples was built. A heatmap of the relative abundances for each compound detected in the wine samples for the two vintages examined is presented in Figure 1. In this case, row-wise normalization (range scaling) was used to aid in the visualization process.

**Figure 2:**
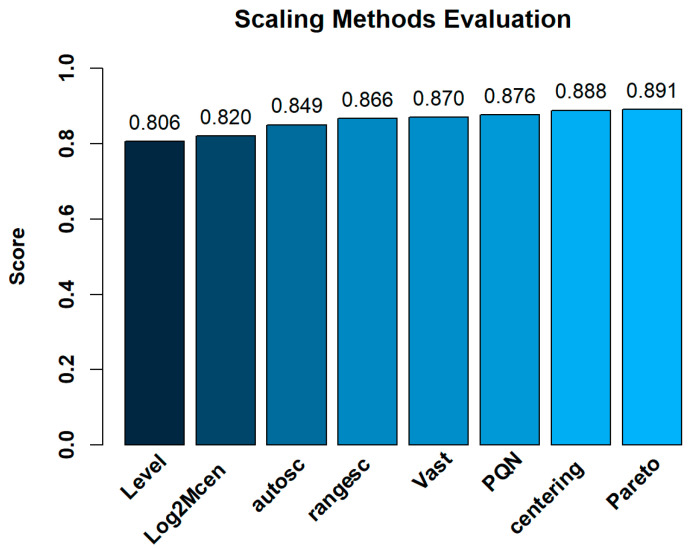
Scaling methods evaluation.

Regarding to the selection of the appropriate scaling method for the subsequent classification and prediction model construction, Pareto scaling showed the highest score (0.891) among the other scaling methods according to ranking score described in Section 3.8 (Figure 2). Table S6 of the ESM presents the complete evaluation results.

#### 2.4.2. Chemometrics

**Figure 3:**
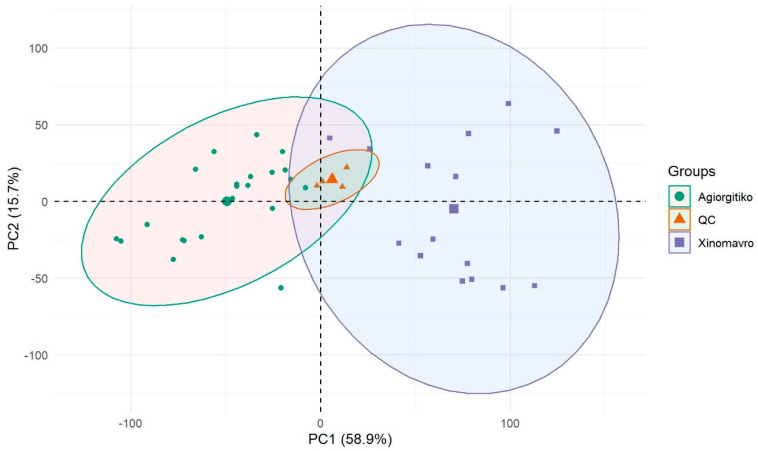
Principal Components Analysis (PCA) score plot showing the unsupervised clustering of red wine samples and QC distribution.

Initially, a Principal Components Analysis (PCA) was performed including all variables in order to reduce the dimensionality of the data and show how they distribute in reduced new variables so called principal components. Although the main purpose of using PCA was to control QC distribution, it was also used to assess whether the choice of supervised classification methods based on singular vector decomposition (SVD) would be successful or not. Figure 3 shows the PCA score plot of red wines. As observed, the first two principal components account for the 74.6% of the model total variance. QC samples are center to the plot and closely clustered demonstrating the good analytical stability maintained. Complete separation among the groups was not achieved as all the variables were considered, including the noisy ones. Moreover, the accumulative value of 74.6% in two latent variables is an indication that methods like PLS-DA or OPLS-DA can be selected for supervised classification.

In order to classify the samples according to grape variety, a removal of certain compounds from the variable list was deemed necessary. Specifically, *m*/*z* ratios of common organic acids including malic, lactic, tartaric, and citric acids were removed from the variable list because seasonal variation, vineyard practices and winemaking protocols have been found to carry a noticeable weight on their abundance [35]. Secondary metabolites, including tyrosol and resveratrol derivatives, were also removed as their variation in the samples was not considered variety driven. The presence of tyrosol derivatives is fermentation induced [36] and can be influenced by different yeast strains while resveratrol derivatives are triggered as a defense response to common vine pathogens [37]. As a consequence, the dataset was reduced to a matrix of 46 samples and 91 variables. 

**Figure 4:**
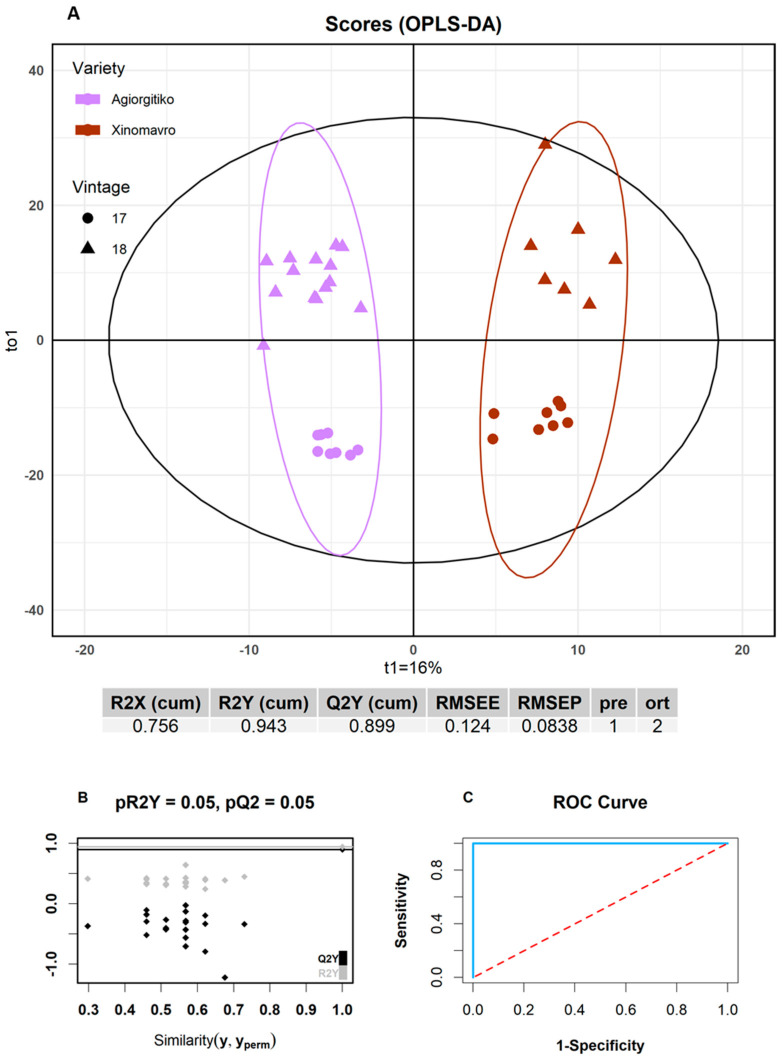
(**A**) Score plot of the OPLS-DA model for the discrimination of *Agiorgitiko* and *Xinomavro* wines based on grape varieties. The number of components and the cumulative R^2^X, R^2^Y and Q^2^Y and RMSEE, RMSEP values are presented below the plot; (**B**) Permutation test: Plot depicting the comparison of the cumulative R^2^Y and Q^2^ values of the model compared with the corresponding values obtained after random permutation of the y response; (**C**) Receiver Operating Characteristics (ROC) curve for the developed OPLS-DA model.

Subsequently, an OPLS-DA model was constructed using 37 out of the 46 samples as a training set, implementing the Kennard stone algorithm for the division. Classification orthogonalization using OPLS-DA was preferred against the classical PLS-DA approach since it provided a better separation of the groups in the score plot even though the models constructed featured the same performance characteristics (Figure S1A,B). The remaining nine samples were used as an external validation test set. Using the Q^2^Y metric and in view of retaining model simplicity, two latent variables were selected, explaining 75.6% of the modeled variance (Figure S1C). The OPLS-DA score plot, shown in Figure 4A, shows that the samples from the two varieties are very well separated. A significant variability was observed within groups. This was attributed to seasonal variation as the two vintages were conducted under contrasting climatic conditions.

Both the training and test set samples were 100% accurately predicted both internally and externally. The values of RMSEE and RMSEP were found to be as low as 0.124 and 0.0838%, respectively. Additionally, a ROC curve (Plot of Sensitivity versus 1—Specificity) was generated for each class, where the area under the curve was calculated as 1.00. Moreover, as seen from the permutation test in Figure 4B, after 20 random permutations, the values of R^2^Y and Q^2^Y did not exceed the ones achieved for the actual model, demonstrating the very high reliability and predictability of the classification model.

**Figure 5:**
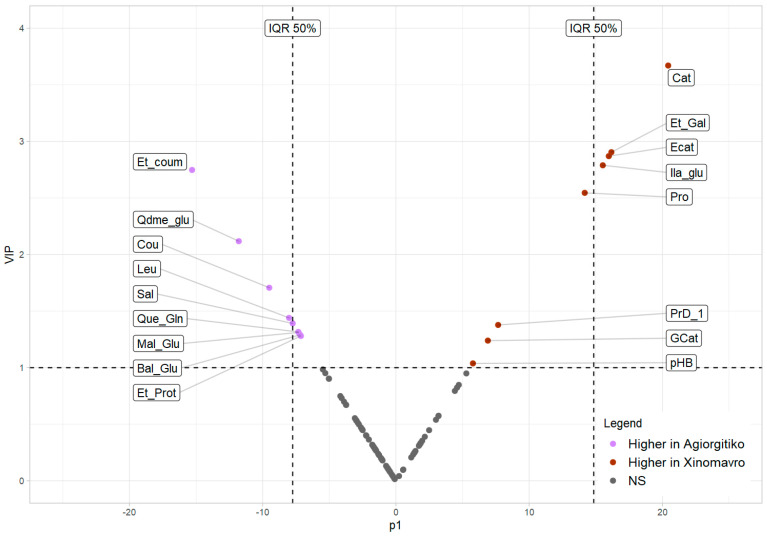
Vplot combining the modeled covariance (p1) and the VIP values of the OPLS DA model. Datapoints in purple are more associated with *Agiorgitiko* wines while the ones in red are more expressed in *Xinomavro* wines. Abbreviations Sal:Salicylic acid; Bal_Glu: Benzyl O-[arabinofuranosyl-(1->6)-glucoside]; Cou: Coutaric acid; Que_Gln: Quercetin.3.glucuronide; Et_coum: Ethyl.coumarate; Et_Prot: Protocatechuic acid ethyl ester; Cat: Catechin; Ecat: Epicatechin, pHB: p-Hydroxybenzaldehyde; Et_Gal: Ethyl.gallate; PrD_1: Procyanidin dimer 1, GCat: Gallocatechin; Pro: L-Proline, Ila_Glu: Indolelactic acid glucoside, Qdme_glu: Quercetin 3,3′-dimethyl ether 4′-glucoside; Leu: (Iso)-Leucine; Mal_Glu: Malvidin 3-O glucoside; IQR50% Optional threshold based on the application of the IQR method to reduce the number of important markers by 50% while retaining model accuracy.

Figure 5 shows a Vplot depicting the most important metabolites contributing to class separation. Variables with VIP values greater than 1 and positive values of p1 are more associated with *Xinomavro* wines while variables with VIP > 1 and negative p1 values are more expressed in *Agiorgitiko* wines. Based on the methodology described in 2.8, seventeen mass features were considered important for the classification model and are presented in Table 2.

An attempt was made to reduce the number of significant mass features using the interquartile range (IQR) method. The application of a 25% cut off and the subsequent evaluation of the models resulted in a lower overall score (Table S8). The reduction of the variables by 50% led to a marginal increase of the initial score (0.871 versus 0.864) and eight significant mass features (Table S7). Although, this approach was not finally adopted in view of not improving the accuracy of classification model, for routine analysis and authenticity problems, it found to be extremely helpful, because it keeps the models accurate, and the quantification and identification task gets simpler.

#### 2.4.3. Identification of Markers

The identification of *m*/*z* values, the selected prioritization method described above, which sought to play a crucial role for the discrimination of Xinomavro and *Agiorgitiko* wines were followed. All the compounds were identified successfully at given acceptable levels. 

**Figure 6:**
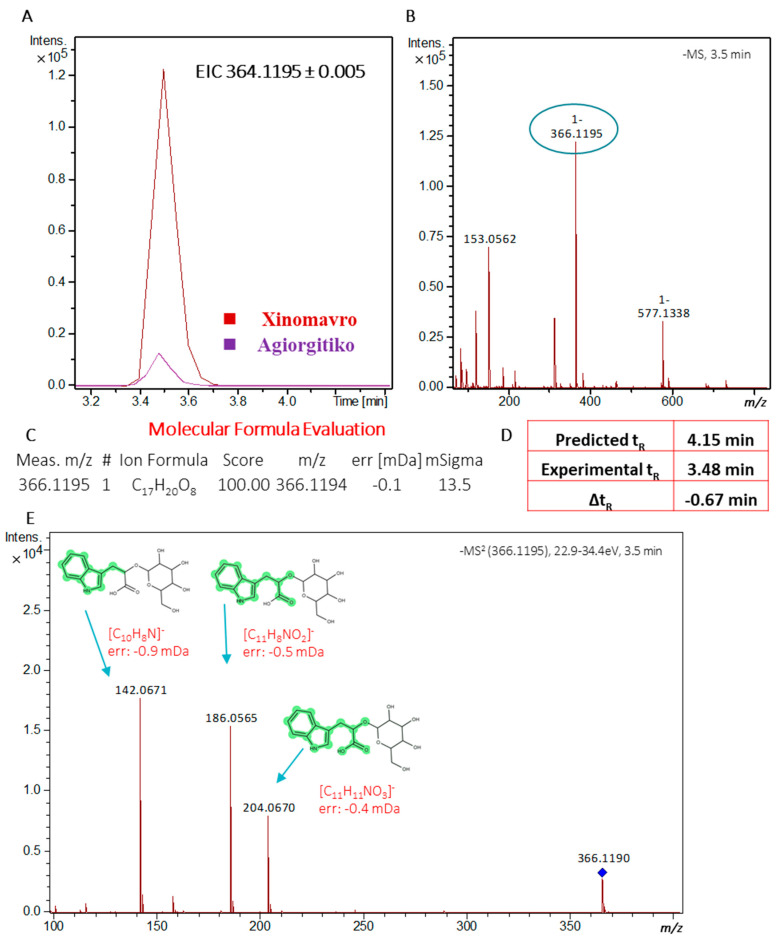
Identification data for the mass feature 366.1195_3.48 min (indolelactic acid glucoside: (**A**) Extracted Ion Chromatogram (EIC) for the given mass (±5 mDa) in *Agiorgitiko* and Xinomavro wine samples; (**B**) fullscan Mass Spectrometry (MS) chromatogram; (**C**) Molecular formula evaluation with Bruker Smart formula manually; (**D**) Comparison between experimental and predicted retention time values based on an in-house QSRR-based retention time prediction model (**E**) MS/MS spectra and corresponding fragments.

One of the most important markers, highly expressed in Xinomavro wines, was *m*/*z* 366.1195, tR = 3.48 min. The presence of the fragment ion with *m*/*z* 204.0670 indicated the loss of a hexose moiety (162.0530 mDa) while the fragments with *m*/*z* values at 186.0560 and 142.0635 were consistent with the fragmentation pattern of indole-3-lactic acid in –ESI mode (Metlin ID: 71). This feature was included in our suspect database as it has been previously reported to exist in wines [38,39]. In the absence of a reference spectrum, the compound was tentatively identified as indolelactic glucoside (Level of Identification of 3). Identification data are provided in Figure 6. Indole-3-lactic acid as well as its glucoside are secondary metabolites produced from tryptophan and their abundance is dependent upon both varietal and climatic conditions [38]. Here for both vintages examined, wines from the Xinomavro variety exhibited higher mean values for both compounds in contrast to *Agiorgitiko* wines.

Moreover, selected *m*/*z* ratios belonging to flavan-3-ols monomers and oligomers were found among the important biomarkers and were more associated with Xinomavro than *Agiorgitiko* wines. (−)-Epicatechin (Figure S2) was identified and quantified using a reference standard (target screening results). (−)-Gallocatechin (Figure S3) (Level 2a) was identified based on reference spectrum match (MoNA ID: MetaboBASE0907) and was distinguished from its isomer (−)-epigallocatechin on the basis of the elution order previously reported under reversed phase chromatographic conditions [40]. Regarding the important mass feature with *m*/*z* 577.1346 eluting at 3.31 min, a molecular formula of C_30_H_26_O_12_ was assigned with a mass accuracy of −0.58 mDa and an isotopic fit of 43.7 mSigma. The compounds fragmentation pattern matched to those of procyanidin dimer. Our suspect database included procyanidin type A and B dimers, however, in the absence of a reference standard, and in order not to speculate between the different procyanidins found in wine [29], the feature was annotated as Procyanidin dimer 1 (Level of identification 3). An isomer with similar fragmentation pattern was also detected at 3.8 min but was not considered important from the classification model. As recently suggested [41], grape variety may have a strong influence on wine proanthocyanidin composition due to the fact that it greatly affects the proportion of skin and seed proanthocyanidins extracted in wine. 

Malvidin 3-O Glucoside was found to be a significant marker for the classification model since it was more expressed in *Agiorgitiko* than *Xinomavro* wines as it has been previously reported [31,41,42]. The tentantive identification of malvidin 3-O glucoside (Figure S4) was based on the characteristic presence of the [M − 2H]^−^ and [M − 2H + H_2_O]^−^ base ions in the MS spectra as suggested earlier [43]. Anthocyanins have an essential role in red wines as they contribute significantly to wine color as well as to reactions affecting the stability and longevity of red wines [34]. In addition, anthocyanins are considered effective varietal markers and malvidin 3-O glucoside is frequently considered the most abundant wine anthocyanin. 

Mass features attributed to two amino acids were found to be important biochemical markers. Amino acids have been previously employed in chemotaxonomical studies of Greek varietal wines [44]. In our study, the *m*/*z* ratio of 130.0874 eluting at tR = 1.92 min, which is putatively identified as (Iso)Leucine (Figure S5) (Level 3), was expressed at high amount in *Agiorgitiko* wines while the opposite was the case for *m*/*z* ratio of 114.0559 eluting at tR = 1.38 min, putatively identified as Proline (Level 2a, Metlin ID: 29).

Specific *m*/*z*’s attributed to phenolic acids and derivatives had a high impact on the classification model. Salicylic acid was identified based on a reference standard. The mass feature detected at *m*/*z* 295.0456_tR = 1.75 min that exhibited fragments corresponding to tartaric (149.0091 mDa) and p-coumaric acid (163.0395) was tentatively identified as the hydroxyl cinnamoyl tartaric acid coutaric acid as previously suggested [39]. Pseudomolecular ions with *m*/*z*’s at 181.0506, 191.0716 and 197.0455 that created [M – H − 28]^−^, [M − H − 72]^−,^ and [M − H − 73]^−^ product ions corresponding to the loss of ethylene, ethylene + CO_2_ and –CO-OCH_2_CH_3_ groups were tentatively identified as ethyl esters of protocatechuic, coumaric and gallic acids, respectively [45].

Two mass features attributed to flavonol derivatives were considered important for the classification model. The mass feature with *m*/*z* 477.0678, tR = 5.00 min was tentatively identified as quercetin 3-glucuronide (Figure S6) (Level 2a). Due to the presence of the fragment ion with *m*/*z* 301.0352 indicating the quercetin aglycon and the presence of *m*/*z* 178.9991 product ion indicating the presence of a glucuronide group. Regarding the mass feature with *m*/*z* 509.1303, tR = 3.3 min, it was tentatively identified as the [M + H2_0_ − H]^−^ ion of Quercetin 3,3′-dimethyl ether 4′-glucoside (Level 2a) as recently suggested from our group [46].

For the mass feature detected at *m*/*z* 401.1439, tR = 4.48 min and expressed high in *Agiorgitiko* wines, the molecular formula of C_18_H_26_O_10_ was assigned with a mass accuracy and isotopic pattern fit of −1.46 mDa and 50.5 mSigma, respectively. This mass feature was included in the in-house database and was putatively identified as benzyl O-[arabinofuranosyl-(1->6)-glucoside]. As a further confirmation step and in the absence of an authentic standard a non-target workflow was implemented in order to verify its tentative identification. 207 candidates sharing the same molecular formula were retrieved from Pubchem [47] and were processed using Metfrag. The list of compounds was further filtered with the use of the QSRR-based retention time prediction model excluding the compounds with retention time error greater than 2 min. This reduced the number of plausible compounds with a Metfrag score above 0.5 to 69. Based on the literature review and in accordance with the predicted retention time the compound was annotated as benzyl 6-O-alpha-L-arabinofuranosyl-beta-D-glucopyranoside (2b) (Pubchem ID: 14682806) which received a Metfrag score of 0.9349 and 6 out of 7 peaks explained. The selected feature shares the same structure with compound with Pubchem ID: 14682805 named as benzyl O-[arabinofuranosyl-(1->6)-glucoside] which was included in our database. The benzyl alcohol derivative has been previously reported present in grapes and alcoholic beverages [48].

## 3. Materials and Methods 

### 3.1. Sample Collections

In this study, 46 fresh monovarietal red wine samples from the native Hellenic Vitis vinifera cv. *Agiorgitiko* and Xinomavro grape varieties were collected. The wines were produced in an industrial scale from wineries that were situated in two important PDO regions, in Southern and Northern Greece (Nemea and Naoussa, respectively) in two successive vintages 2017–2018 (Table S1). The samples were both tank samples bottled under nitrogen atmosphere in 0.75 L bottles and commercially available wines. A table depicting sample origin and vintage year can be found in Table S1 of the Electronic Supplementary Material (ESM).

### 3.2. Chemicals and Reagents

All standards and reagents were LC-MS grade and were used without any further treatments. Authentic standards of 2,5-Dihydroxybenzoic acid (gentisic acid), 3,4-dihydroxybenzoic acid (protocatechuic acid), 4-hydroxybenzoic acid, cinnamic acid, epicatechin, eriodictyol, ferulic acid, gallic acid, myricetin, p-coumaric acid, pinoresinol, quercetin, resveratrol, rosmarinic acid, salicylic acid, syringic acid, taxifolin, and vanillic acid were purchased from Sigma-Aldrich (Stenheim, Germany). Apigenin, caffeic acid, catechin, ethyl vanillin (internal standard, IS), galangin, genistein, naringenin, tyrosol, and vanillin were acquired from Alfa Aesar (Karlsruche, Germany), whereas hydroxytyrosol and luteolin were obtained from Santa Cruz Biotechnology (Santa Cruz, CA, USA). 

2-Propanol (LC–MS grade) and methanol (MeOH) (LC–MS grade) were purchased from Fisher Scientific (Geel, Belgium) and Merck (Darmstadt, Germany), respectively. Formic acid 99%, ammonium acetate and sodium hydroxide monohydrate for trace analysis ≥ 99.9995% were all obtained from Fluka (Buchs, Switzerland). Ultrapure water (18.2 MΩ resistivity) was provided by a Millipore Direct-Q UV purification System (Millipore, Bedford, MA, USA). The wine samples were filtered using regenerated cellulose syringe filters (RC filters, pore size 0.2 μm, diameter 15 mm) purchased from Phenomenex (Torrance, CA, USA).

### 3.3. Preparation of Standard Stock Solutions

Standard stock solutions were prepared by dissolving appropriate amounts of each individual analyte in pure MeOH to a final concentration of 1000 μg/mL and were stored at −20 °C in amber glass bottles to prevent photodegradation. Ethyl vanillin, a compound that is not present in wine, was used as an internal standard (IS) in order to correct potential drift of the analytical signal throughout the batch. A stock solution of the IS was also prepared in MeOH at a concentration of 200 μg/mL. Working mix solutions containing all target analytes were prepared in order to construct standard calibration curves with concentrations ranging from 0.1 to 20 mg/L. The working solutions were prepared by gradient dilution of the stock solutions in methanol/water (1:1, *v*/*v*). The final concentration of the IS used in the working solutions was set 2 mg/L. 

### 3.4. Sample Preparation

Before the analysis, each bottle of wine was uncorked under nitrogen atmosphere and approximately 3 mL of the sample were filtered with 0.22 μm RC syringe filters. An aliquot of 990 μL of filtered wine was mixed with 10 μL of the IS stock solution and transferred to 2 mL amber autosampler vials and they were kept at 4 °C until analysis. The samples were then directly injected in UHPLC-QToF-MS system.

A quality control (QC) sample was also prepared by mixing same-volume aliquots of all wine samples (0.5 mL of each). This QC sample was analyzed recurrently throughout the batch in order to assess sample stability during analysis as well as inter/intra-day variability. 

### 3.5. UHPLC–QToF-MS Analysis 

The analysis of wine samples was conducted using an UHPLC system (Dionex UltiMate 3000 RSLC, Thermo Fisher Scientific, Germany) equipped with a solvent rack degasser, a binary pump with solvent selection valve (HPG-3400) and an auto-sampler coupled via an Electrospray Ionization (ESI) interface to an Ultra high Resolution Quadrupole Time of Flight Mass spectrometer (Maxis Impact, Bruker Daltonics, Bremen, Germany). The use of UHPLC systems enhances the chromatographic resolution and peak capacity while reducing the analysis time by using smaller particles in the stationary phase.

The chromatographic analysis was operated with a reversed phase (RP) separation method using an Acclaim RSLC 120 C18 column (2.1 × 100 mm, 2.2 μm) from Thermo Fischer Scientific (Dreieich, Germany) preceded by a ACQUITY UPLC BEH C18 1.7 μm, VanGuard Pre-Column from Waters (Dublin, Ireland). The column temperature was 30 °C. The mobile phase consisted of solvent A (5 mM ammonium acetate in 90:10, *v*/*v*, H2O/MeOH) and solvent B (5 mM ammonium acetate in MeOH). The gradient elution program started with 1% of solvent B (flow rate 0.2 mL min^−1^), which then reached to 39% in the next 3 min with the same flow rate and finally to 99.9% (flow rate of 0.4 mL min^−1^) in another 11 min. These conditions were kept constant for 2 min with a flow rate of 0.48 mL min^−1^ and afterwards the column was re-equilibrated restoring the initial conditions. 

The MS analyzer was operated in negative electrospray ionization mode with nebulizer gas pressure of 2.0 bar (N2), dry gas flow of 8.0 L/min, dry gas temperature of 200 °C, capillary voltage of 3500 V, end plate offset of −500 V. Injection volume was set to 5 μL. MS data were recorded over a range of *m*/*z* from 50 to 1000 with a scan rate of 2 Hz in 2 scan modes; Bruker broadband collision-induced dissociation (bbCID) mode, which is a data independent acquisition mode (DIA) and AutoMS (data dependent) mode. In bbCID both the precursor and product ions spectra (MS and MS/MS) are obtained within a single injection, by using two different collision energies (4 eV and 25 eV, respectively). In AutoMS, full scan MS spectra are obtained and the five most abundant precursor ions for each MS scan are isolated and fragmented with a predefined collision energy, providing compound specific MS/MS spectra. In the case of masses with low intensity in which no MS/MS information were recorded via AutoMS mode, an inclusion list including the suspected masses was created.

An external calibration of the QToF mass spectrometer was carried out daily by infusing a sodium formate calibrant solution, consisting of 10 mM sodium formate clusters in a mixture of water: isopropanol (1:1, *v*/*v*) before analysis. Moreover, the same calibrant solution was injected automatically at the beginning of each chromatographic run and the segment of 0.1–0.25 min was reserved for internal calibration. The theoretical exact masses of fourteen calibration ions with formulas Na(NaCOOH)_x_, where x equals 1 to 14, in the range of 50–1000 Da were used for calibration. Bruker high-precision calibration algorithm enabled the calibration of data files. The apparatus provided a typical resolving power (Full width at half maximum, FWHM) of 19,000–24,000 during calibration at *m*/*z* 226.1593, 430.9137, and 702.8636. 

### 3.6. Method Validation

Method linearity, limits of detection (LOD) and quantification (LOQ), precision, and matrix effects were evaluated. For the assessment of method linearity, 7-point calibration curves were prepared for all target analytes within the expected concentration range (0.1 to 20 mg/L) with the use of the working solutions of the standard compound mixtures. The curves were prepared by plotting the ratios of analyte peak area divided to the peak area of the internal standard versus the concentration of the analyte. Linear regression analysis was used for the calculation of slope, intercept, and correlation coefficient data. Simultaneously, matrix matched calibration curves were prepared by spiking one pool red wine sample with the target analytes in order to evaluate the methods’ matrix effects. For the compounds already present in the pool samples, the amount of the sample was subtracted before plotting the matrix-matched calibration curve. 

The matrix factor was calculated by dividing the slope of the matrix matched curve to the slope of the calibration curves of the analytes in pure solvent. The matrix effect was calculated according to the following equation:

%Matrix Effect = (Matrix Factor − 1) × 100(1)

Instrumental precision was determined by repeatedly injecting standard compound mixtures (*n* = 5) at two concentration levels (0.5 and 5.0 mg/L) and evaluating the relative standard deviation (RSD%) of the peak areas of the detected compounds. The method LODs and LOQs were calculated as the lowest analyte concentrations that could be detected in wine samples with a signal-to-noise ratio of 3 and 10, respectively. The quantification of the phenolic compounds in all wine samples was performed using an external standard calibration method through reference standard calibration curves.

### 3.7. Screening Strategies

#### 3.7.1. Target Screening 

Our study was initially targeted at the determination of specific phenolic compounds that could potentially act as biomarkers, enabling wine characterization and varietal discrimination. Hence, a database was constructed containing 28 phenolic compounds belonging to various chemical classes. Identification and quantification of target compounds was performed using authentic standards of these compounds available at the lab. The identification workflow for the target compounds was performed according to Dasenaki et al., 2019 [46]. The information included in the database comprised of the analyte molecular formula, the *m*/*z* value for the main adduct form of [M − H]^−^, the experimental retention time (tR) in min and the MS/MS fragments for each target analyte. The aforementioned information is presented in Table S2 of the ESM. 

Following data acquisition, the raw data were processed with Data Analysis 4.4 and TASQ 1.4 software packages (Bruker Daltonics Bremen, Germany). The target compounds were evaluated and confirmed after meeting specific criteria regarding mass accuracy (<2 mDa), retention time tolerance (<0.2 min), isotopic pattern fitting (Bruker’s mSigma value with values ≤ 50), peak area (>800), peak intensity (>200) and MS/MS fragmentation. Extracted Ion Chromatograms (EICs) of the precursor ions were created for all target compounds that exceeded peak area and peak intensity values and were evaluated in all samples. 

#### 3.7.2. Suspect Screening Strategy

An in-house suspect screening database, containing 420 compounds putatively present in grape and wine, was built by employing information retrieved from several open access databases such as FooDB (http://foodb.ca; accessed on 10 April 2021), Phenol Explorer (http://phenol-explorer.eu; accessed on 1 April 2021), GrapeCyc (https://www.plantcyc.org, accessed on 10 April 2021) and from the study of scientific literature. The suspect database included information necessary for identification of detected compounds such as the putative molecular formula, MS/MS fragments and predicted retention times. Five qualifier ions or frequently observed MS/MS fragments across different collision energies were collected from EU MassBank (www.massbank.eu; accessed on 10 April 2021), mzcloud (https://www.mzcloud.org/; accessed on 10 April 2021), MoNA (https://mona.fiehnlab.ucdavis.edu; accessed on 10 April 2021), and Metlin (http://metlin.scripps.edu; accessed on 10 April 2021). The predicted retention time values were added in the suspects list using Quantitative Structure-Retention Relationships (QSRR) retention time prediction models [49]. These models have been used already in foodomics and HRMS based screening strategies [50,51].

After building the database, a robust workflow for the suspect screening of compounds in wine samples was used according to Gago-Ferrero et al. (2015) [52]. A tentative identification of the compounds was performed according to specific thresholds set regarding: mass accuracy of monoisotopic peak(<5 mDa); peak area (>2000) and ion intensity(>800 counts); isotopic fit values(mSigma < 100); peak score > 4 (peak score = peak area to peak intensity ratio >4). Moreover, retention times of all identified compounds inside the applicability domain of the model should not exceed a 1.8 min window between the predicted and experimental retention time values [53]; and at least one characteristic MS/MS fragment (base ion) included in the suspect database should be detected and interpreted. For compounds where MS/MS data were unavailable, in silico fragmentation tools CFM-ID [54] was used and MetFrag [55] was applied for ranking candidates based on their explained MS/MS fragments. To communicate identification confidence level regarding the suspect screening results, identification levels were assigned according to the classification proposed by Schymanski et al. (2014) [56]. The presence of false positives due to contaminations or analytical procedural blank was evaluated by analyzing a pure solvent sample every 10 sample injections.

### 3.8. Data Processing and Chemometrics

Data normalization and scaling were necessary prior to higher order statistical analysis. HRMS peak lists were normalized to the peak area of the IS and then scaled using Pareto scaling (i.e., mean-centering the variables and dividing by the square root of the standard deviation). Pareto scaling was selected after comparing different scaling methods and using a previously developed score [57] from our group based on statistical model performance. To assess the internal and external accuracy of the supervised classification model, the datasets were divided by Kennard stone algorithm [58] into a training and a test set with a ratio of 80% to 20%, respectively. 

Multivariate classification methods were then employed to discriminate red wines based on their variety and reveal characteristic varietal markers. Initially, Principal Component Analysis (PCA) was conducted with the use of the R package “*factoextra*” [59] in order to evaluate QC distribution. Orthogonal Projection to Latent Structure-Discriminant Analysis (OPLS-DA) was employed for pairwise markers discovery and discrimination between wines samples. The analysis was performed with the use of the R package “*ropls*” [60]. Latent variable selection was performed on the basis of evaluating the change in the predictive performance of the model estimated by cross-validation (Q^2^Y metric). An OPLS-DA score plot was generated to depict class discrimination and Variable Importance in Projection (VIP) values were also obtained. 

The model was validated both internally, using leave-one-seventh-out cross validation, and externally, using nine samples of both varieties as a test set. Predicted values for the class membership of each sample were generated using the max dist function from the “*ropls*” R package. Consequently, specificity, sensitivity and total accuracy were calculated and a Receiver Operating Characteristics (ROC) curve was prepared.

Model performance was assessed by R^2^X (cumulative modeled variation explained by the two latent variables), R^2^Y (goodness of fit), Q^2^Y (predictive performance of the model estimated by cross-validation) as well as RMSEE (Root Mean Square Error of Estimation (for training set) and RMSEP (Root Mean Square Error of Prediction (for test set). The randomness and robustness of the model was also evaluated by a permutation test (*n* = 20) of cumulative R^2^Y and Q^2^ values [60]. 

Variables featuring Variable in Projection (VIP) values > 1 were considered as important [61]. Values of modeled covariance p[1] and modeled correlation p(corr)[1] were estimated for all variables. The VIP threshold above was applied in order to discover significant markers. A Vplot combining p[1] values in the abscissa and VIP values in the ordinate was prepared. Variables with higher absolute p[1] values have a larger contribution on the variance between the groups. Consequently, data points that fall on the upper left and upper right corner contributed significantly to the model and may be considered as important biomarkers separating the classes.

## 4. Conclusions

A novel HRMS metabolomics approach was developed for the profiling and authentication of wine samples derived from the emblematic Greek *Agiorgitiko* and Xinomavro grape varieties. Target screening strategy was used to determine and quantify 28 wine metabolites belonging to different classes. The subsequent application of a smart suspect screening workflow was proved efficient in detecting and putatively identifying 86 additional compounds present in the wine samples, providing a thorough chemical characterization. Mass spectrometric data derived from both target and suspect screening were used for advanced chemometrics. A smart selection of scaling methods was adopted. Chemometrics using OPLS-DA led to a robust, accurate and double validated classification and prediction model, successfully classifying the wine samples according to grape variety. Seventeen compounds were suggested as characteristic authenticity markers responsible for the discrimination between the wine samples. The use of the IQR method, though not finally adopted, and its application on the O-PLS-DA model, showed great potential in reducing the number of marker compounds to be determined while retaining the model classification accuracy. This approach can be particularly helpful in future authenticity studies or routine analysis. This study provides a valuable insight on the profile of wines from the *Agiorgitiko* and Xinomavro grape varieties as well as a robust and reliable workflow which can be employed in wine authentication.

## Figures and Tables

**Table 1:** Concentrations of target compounds detected in the red wine samples.

Group	*Agiorgitiko* (*n* = 27)		*Xinomavro* (*n* = 19)		All Samples (*n* = 46)	
	Concentration (mg/L)					
	mean	range	Mean	range	mean	range
4-Hydroxybenzoic acid	0.52 ^a^	0.14–1.8	0.34 ^a^	0.13–0.71	0.44	0.13–1.8
Caffeic acid	2.4 ^a^	0.98–5.4	2.4 ^a^	0.79–5.1	2.4	0.79–5.4
Catechin	27 ^a^	18–38	65 ^b^	44–83	43	18–83
Epicatechin	15 ^a^	8.0–26	38 ^b^	18–59	24	8.0–59
Eriodictyol	traces		traces		traces	
Ferulic acid	0.22 ^a^	<LOD–0.66	0.34 ^a^	0.24–0.47	0.27	<LOD–0.66
Gallic acid	26.4 ^a^	11.1–46	43 ^b^	27–64	33	11–64
Gentisic acid	0.481 ^a^	0.12–0.92	0.43 ^a^	0.11–0.71	0.46	0.11–0.90
Hydroxytyrosol	0.992 ^a^	0.72–1.4	2.7 ^b^	1.7–3.9	1.7	0.72–3.9
Luteolin	traces		traces		traces	
Myricetin	2.4 ^a^	0.86–4.5	4.5 ^b^	1.1–10	3.3	0.86–10
Naringenin	0.10 ^a^	<LOD–0.16	0.072 ^b^	<LOD–0.13	0.091	<LOD–0.16
p-Coumaric acid	1.80 ^a^	0.65–5.45	1.02 ^b^	<LOD–4.0	1.48	<LOD–5. 5
Protocatechuic acid	3.0 ^a^	1.0–7.7	1.3 ^b^	0.28–2.4	2.4	0.28–7.7
Quercetin	2.7 ^a^	1.1–5.3	6.2 ^b^	0.29–10	4.2	0.29–10
Resveratrol	1.4 ^a^	0.21–2.6	0.78 ^a^	0.41–1.7	1.1	0.21–2.6
Salicylic acid	0.67 ^a^	0.13–1.5	0.26 ^b^	0.17–0.51	0.50	0.13–1.5
Syringic acid	4.7 ^a^	0.98–7.4	2.1 ^b^	1.2–3.7	3.6	0.98–7.4
Taxifolin	0.10 ^a^	<LOQ–0.17	0.11 ^a^	0.060–0.20	0.11	<LOQ–0.20
Tyrosol	28 ^a^	13–43	43 ^b^	17–74	34	12–74
Vanillic acid	1.9 ^a^	0.81–3.8	0.99 ^b^	0.6–1.8	1.5	0.60–3.8
Vanillin	traces		traces		traces	

Different letters on the mean values of each compound denote significant differences between the varieties (*Wilcoxon–Mann–Whitney test*, p.adj < 0.05). traces: concentrations between LOD and LOQ values in random samples.

**Table 2:** List of discriminant VIP markers detected in red wines from different grape varieties.

	Molecular Formula	Proposed Compound	Class	p1	pcorr1	VIP
1	C_15_H_14_O_6_	Catechin	Flavan-3-ols	20.44	0.51	3.67
2	C_9_H_10_O_5_	Ethyl gallate	Benzoates	16.17	0.31	2.90
3	C_15_H_14_O_6_	Epicatechin	Flavan-3-ols	15.98	0.54	2.87
4	C_17_H_21_NO_8_	Indolelactic acid glycoside	Amines	15.53	0.79	2.79
5	C_11_H_12_O_3_	Ethyl coumarate	Cinnamates	−15.30	−0.64	2.75
6	C_5_H_9_NO_2_	L-Proline	Amino acids	14.17	0.32	2.54
7	C_23_H_26_O_13_	Quercetin 3,3′-dimethyl ether 4′-glucoside	Flavonols	−11.79	−0.70	2.12
8	C_13_H_12_O_8_	Coutaric acid	Cinnamates	−9.50	−0.53	1.71
9	C_6_H_13_NO_2_	(Iso)leucine	Amino acids	−8.02	−0.45	1.44
10	C_7_H_6_O_3_	Salicylic acid	Benzoates	−7.75	−0.50	1.39
11	C_30_H_26_O_12_	Procyanidin dimer 1	Flavan-3-ols	7.67	0.35	1.38
12	C_21_H_18_O_13_	Quercetin 3-glucuronide	Flavonols	−7.33	−0.48	1.32
13	C_23_H_24_O_12_	Malvidin 3-0 Glucoside [M-2H]	Anthocyanins	−7.30	−0.66	1.31
14	C_18_H_26_O_10_	Benzyl O-[arabinofuranosyl-(1->6)-glucoside]	Benzyl alcohol derivatives	−7.19	−0.44	1.29
15	C_9_H_10_O_4_	Protocatechuic acid ethyl ester	Benzoates	−7.13	−0.45	1.28
16	C_15_H_14_O_7_	Gallocatechin	Flavan-3-ols	6.90	0.48	1.24
17	C_7_H_6_O_2_	p-Hydroxybenzaldehyde	Benzaldehydes	5.78	0.55	1.04

## Data Availability

The data presented in this study are available in article and supplementary material.

## Supplementary Materials

The following are available online. Table S1—Number and origin of wine samples; Table S2—Target compound information; Table S3—Target screening method validation; Table S4—Evaluation of Normality Assumptions; Table S5—Identified Compounds; Table S6—Scaling Methods Evaluation; Table S7—IQR Method Evaluation; Table S8—IQR Compound list; Figure S1—Comparison of OPLS-DA and PLS-DA models; Figure S2—Epicatechin Identification data; Figure S3—Gallocatechin Identification data; Figure S4—Malvidin 3-O Glucoside Identification data; Figure S5—(Iso)Leucine Identification data; Figure S6—Quercetin 3-O Glucuronide Identification data.

## Sample Availability

Samples of the compounds are not available from the authors.
